# Broad Evidence Triangulation Should Be Established for a Valid and Robust Causal Relation Between Air Pollution and Health Outcome

**DOI:** 10.1111/cns.70207

**Published:** 2025-01-03

**Authors:** Tongyu Gao, Hao Zhang, Yu Yan, Yuxin Liu, Zhou Jiang, Ping Zeng

**Affiliations:** ^1^ Department of Biostatistics, School of Public Health Xuzhou Medical University Xuzhou Jiangsu China; ^2^ Department of Epidemiology and Biostatistics, School of Public Health, Tongji Medical College Huazhong University of Science and Technology Wuhan China; ^3^ Jiangsu Engineering Research Center of Biological Data Mining and Healthcare Transformation Xuzhou Medical University Xuzhou Jiangsu China

## Abstract

This letter aims to provide valuable insights into broader evidence triangulation (i.e., a well‐designed primary association analysis followed by elaborate approaches to control residual confounding effects from various design and modeling perspectives) for clarifying the association between air pollutants and health outcomes. It also highlights the importance of selecting appropriate instrumental variable for instrument‐based causal modeling, emphasizing that all causal questions can be effectively addressed within the Mendelian randomization framework.

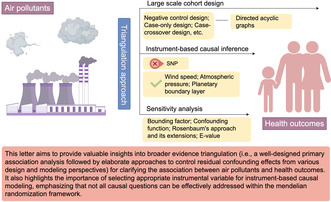

Dear Editor,

We are writing this letter to express our interest regarding a recently published paper [1]. One of the main goals of that work was to mitigate residual confounding and reverse causation by implementing Mendelian randomization (MR) to evaluate the causal effect of long‐term exposure to particulate matter (e.g., PM_2.5_) on amyotrophic lateral sclerosis with single nucleotide polymorphisms (SNPs) serving as instrumental variables (IVs), which were available from genome‐wide association study (GWAS) summary statistics of the UK Biobank.

However, after thoroughly reviewing the MR methodology there, we are concerned about significant design flaws that can undermine the validity of this research. Specifically, we raise doubts about the effectiveness of leveraging SNPs as IVs for air pollutants because the existence of specific SNPs influencing such an external exposure is implausible from a biological standpoint. In fact, air pollutants of the UK Biobank were inferred via residential home addresses rather than individual measurements through the Land Use Regression model [2], which implies that any genetic indicators of air pollutants just act as proxies for socioeconomic status [3], rather than reflecting inherent biological mechanisms [4]. Consequently, this causes our concerns about artificial correlations between the selected IVs and air pollutants, which violates the relevance assumption—one of the three core conditions of a valid MR study, and leads to statistically robust yet biologically inconsequential results even if the MR analysis was correctly performed. Similarly, the same concern of biological rationality also emerges when the transcriptome‐wide association study analysis was conducted to identify air pollutant‐related genes in this work.

Ideally, the combination of cohort study results with causal inference techniques offers a robust evidence triangulation framework to understand the causal connection between air pollutants and a health outcome of interest. As mentioned by [1], the essential objective of carrying out MR is to scrutinize residual or unknown confounders in an observational causal inference. Over the past few decades, remarkable advancements in causal inference techniques have promoted many successful applications, enhancing the ability of effectively handling confounding effects in observational studies. For known and measurable confounders, well‐developed statistical methods, including traditional ways such as adjustment, stratification, restriction, matching and regression, as well as non‐traditional manners such as disease risk score, propensity score matching, and inverse probability weight, have been widely employed. However, these approaches are powerless against unmeasured confounders.

In practice, it is generally impossible to completely identify and measure all potential confounders. To address unknown or unmeasured confounders (i.e., residual confounding), IV‐based methods have been exploited for a long time, with MR being a recently fruitful application of such approaches facilitated by public availability of summary statistics from large‐scale GWASs. But when it comes to external environmental exposures (e.g., air pollutants) that are not genetically determined, SNP is no longer a biologically appropriate choice as IV. To examine the causal connection of long‐term exposure to air pollution with health outcomes, recent studies have successfully utilized other types of suitable IVs other than SNPs for air pollutants, including wind speed, atmospheric pressure and planetary boundary layer (PBL) [5].

On the other hand, sensitivity analysis techniques (e.g., bounding factor [6], confounding function [7], Rosenbaum's approach and its extensions, and the E value [8]), although not yet received sufficient attention in epidemiology, have been proposed to provide supporting information regarding the determination of the exposure‐outcome association. These methods serve as complementary tools to primary association analyses and seek to answer the question of whether unmeasured confounding factors can fully or partially explain the exposure‐outcome effect identified in an observational study.

Last but not least, novel study designs, including the negative control design, case‐only design, case‐crossover design, case‐time‐control design, self‐controlled case series, and prior event rate ratio adjustment, are encouraged to be employed for the control of unknown confounders in nonexperimental or observational research if possible. Meanwhile, directed acyclic graphs also play a key role in mapping out the relationships among various variables in a clear and visual format, providing a structured and transparent way to identify and understand the causal connections and potential biases in the data.

In conclusion, we hope that our comments provide valuable insights of a broader evidence triangulation (i.e., a well‐designed primary association analysis followed with elaborate treatments of controlling residual confounding effects from various design and modeling perspectives) for studies aiming to clarify the association between air pollutants and health outcomes. To illustrate this, readers are referred to [9] for an excellent example of such research comprehensively using large‐scale prospective cohort analysis, alternative IVs and negative control design under the evidence triangulation framework. It also reminds us that the choice of IVs is crucial for instrument‐based causal modeling and not all causal questions can appropriately be handled within the MR paradigm [10].

## Conflicts of Interest

The authors declare no conflicts of interest.

## Data Availability

The authors have nothing to report.
